# Non-24-Hour Sleep–Wake Rhythm Disorder in the Totally Blind: Diagnosis and Management

**DOI:** 10.3389/fneur.2017.00686

**Published:** 2017-12-18

**Authors:** Maria Antonia Quera Salva, Sarah Hartley, Damien Léger, Yves A. Dauvilliers

**Affiliations:** ^1^AP-HP Hôpital Raymond Poincaré, Sleep Unit, Physiology Department, Versailles Saint-Quentin-en-Yvelines University, Garches, France; ^2^Université Paris Descartes, Sorbonne Paris Cité, APHP, Hôtel-Dieu, Centre du Sommeil et de la Vigilance et, Paris, France; ^3^Sleep-Wake Disorders Center, Department of Neurology, Gui de Chauliac Hospital, National Reference Network for Narcolepsy, INSERM U1061, Montpellier, France

**Keywords:** circadian, melatonin, blind, light, clock, tasimelteon, sleep

## Abstract

Several aspects of human physiology and behavior are dominated by 24-h circadian rhythms with key impacts on health and well-being. These include mainly the sleep–wake cycle, vigilance and performance patterns, and some hormone secretions. The rhythms are generated spontaneously by an internal “pacemaker,” the suprachiasmatic nuclei within the anterior hypothalamus. This master clock has, for most humans, an intrinsic rhythm slightly longer than 24 h. Daily retinal light exposure is necessary for the synchronization of the circadian rhythms with the external 24-h solar environment. This daily synchronization process generally poses no problems for sighted individuals except in the context of jetlag or working night shifts being conditions of circadian desynchrony. However, many blind subjects with no light perception had periodical circadian desynchrony, in the absence of light information to the master clock leading to poor circadian rhythm synchronization. Affected patients experience cyclical or periodic episodes of poor sleep and daytime dysfunction, severely interfering with social, academic, and professional life. The diagnosis of Non-24 Sleep–Wake Rhythm Disorder, also named free-running disorder, non-entrained disorder, or hypernycthemeral syndrome, remains challenging from a clinical point of view due to the cyclical symptoms and should be confirmed by measurements of circadian biomarkers such as urinary melatonin to demonstrate a circadian period outside the normal range. Management includes behavioral modification and melatonin. Tasimelteon, a novel melatonin receptor 1 and 2 agonist, has demonstrated its effectiveness and safety with an evening dose of 20 mg and is currently the only treatment approved by the FDA and the European Medicines Agency.

## Key Messages

- Non-24-h sleep–wake rhythm disorder is an orphan disease in the general population but common in the totally blind.
- Lack of light information to the suprachiasmatic nucleus, the central biological clock leads to the patient’s biological rhythms following their innate period, which is often longer than 24 h.
- This circadian drift leads to cyclical sleep disturbances and daytime sleepiness which pose a major handicap.
- Accurate diagnosis and treatment with both behavioral therapy and medication targeting circadian rhythms, such as melatonin and melatonin agonists, improves symptoms.

## Introduction

Most biological processes display an endogenous, entrainable oscillation with a period of about 24 h, a rhythm driven by a circadian clock named “circadian rhythm” (from circa = around and dies = a day) (1, 2). The molecular circadian clock involves major genes: period (Per1/2/3), Clock, Cryptochrome (Cry1/2), Bmal1, and nuclear orphan receptors (Rev-erbα/β and Rorα/β/γ) with transcription loops that generate the observed rhythms (3–5). The circadian clock mechanism involves a transcriptional feedback loop in which CLOCK and BMAL1 stimulate the transcription of the Period and Cryptochrome genes. Oscillating transcription loops are present in cells in the brain and peripheral organs, and this multi-oscillatory network is organized by the master biological clock, located in the suprachiasmatic nuclei (SCN) (3), leading to an endogenous rhythm. The time taken to complete one cycle of the circadian rhythm is defined as the circadian period. Under stringent conditions, the periodicity of the circadian clock has been estimated to be closer to 24 h (i.e., 23.8–25.1 h) with the clock being reset every morning by exposure to daylight (6). An individual who is entrained to the 24-h day/night cycle has a circadian period set at exactly 24 h. If a person’s non-entrained period is 24.5 h, the sleep/wake cycle and other rhythms will cycle every 24.5 h without any entraining signal, and the subject will go to sleep and wake up 0.5 h later each day. This situation is called “free running,” “hypernycthemeral,” or non-24-h sleep–wake rhythm disorder (non-24 SWRD). Although many environmental signals or “zeitgebers” can entrain circadian rhythms, by far the most important is light. The importance of light in synchronizing rhythms is shown experimentally by the effects of constant dim light conditions in sighted individuals but also by the higher risk of non-24-h rhythm in patients who are completely blind.

Melatonin (*N*-acetyl-5-methoxytryptamine) discovered in 1958 is synthesized by the pineal gland and inhibited by light (7). Melatonin secretion is highly sensitive to changes in the photoperiod length (i.e., light and dark duration exposures) with both circadian and seasonal variations, and acts as a “photo neuroendocrine transducer” providing photoperiod information to the organism. Melatonin synthesis is, thus, controlled by light and ocular light exposure is the key stimulus.

The mammalian retina contains rods, cones, and retinal ganglion cells expressing the photopigment melanopsin (mRGCs) which play a key role in mammals in circadian photoentrainement. mRGC projections extend across the dorsolateral geniculate nucleus up to the pretectal nucleus in the midbrain and are known to drive the pupillomotor light reflex. Photoreceptors drive the circadian photoentrainment across different light intensities (8, 9) and are particularly sensitive to short wavelength blue light in the 460 nm spectrum. mRGCs may survive with complete rod and cone loss in people with advanced retinal degeneration and may contribute to assessing brightness and supporting vision (10–12).

Light that reaches the retina photoreceptors activates SCN *via* the retinohypothalamic tract with signals traveling to the cervical superior ganglion and the pineal gland that responds by inhibiting melatonin synthesis. A regulatory feedback loop involving melatonin modulates SCN activity. Melatonin levels peak during the night, decrease at the end of the night and remain low during daytime, rising again as darkness falls. The nocturnal peak is associated with maximum sleep propensity, and a nadir in the associated rhythms of core body temperature, vigilance, mental performance, and metabolisms.

Melatonin acts *via* the MT1 and MT2 receptors that are G protein-coupled receptors with seven transmembrane domains (13). MT1 and MT2 receptors are widely distributed, and are found in the retina, SCN, other brain regions (i.e., preoptic area, olfactory bulb, prefrontal and cerebellar cortices, hippocampus, basal ganglia, substantia nigra, ventral tegmental area, nucleus accumbens…), peripheral organs (i.e., kidney, adrenal gland, intestine, stomach, heart, lung, skin, testis, ovary), but also in macrophages, adipocytes, and blood platelets (14, 15). MT1 and MT2 mediate the chronobiotic and sedative effects of melatonin (16). In the SCN, MT2 receptors reset the circadian clock while MT1 receptors suppress neuronal firing. A third receptor, MT3, has been identified as an enzyme, quinone reductase. It is widely distributed in mammals (17, 18) but relationships between the physiological functions of melatonin and quinone reductase are unclear. Overall, melatonin has a key component in the regulation of the circadian rhythms.

In totally blind people, circadian processes may become desynchronized due to the absence of light input into the circadian clock. This leads to an abnormal phase angle between the circadian clock and sleep–wake behavior and as a result alters the pattern of alertness, mood, and performance across the day, which interferes with social, academic, and professional life. Most totally blind people with a non-24 SWRD feel it to be more disabling than their blindness (19).

We searched PubMed, Web of Science, and Scopus for all articles reporting non-24 SWRD in blind patients and its management in March 2017 without a language restriction. We also checked the references of all relevant studies and review articles to identify additional sources.

## Epidemiology of Sleep–Wake Cycle Disturbances in Blind Patients

The prevalence of blindness is difficult to establish as there is no uniform definition. The global Burden of Disease study in 2010 estimated that 32.4 million people were blind (20). The prevalence of blindness in US is considered to be close to 1 million for 450 million inhabitants (21), of whom 20% are totally blind (i.e., without light perception). It has been estimated that 120,000 patients in the EU are totally blind.

Several surveys showed a high incidence of insomnia among blind people (21–24). Miles and Wilson (23) earlier reported that 76% of blind subjects (*n* = 50) had sleep/wake disorders, and 40% of them recognized that symptoms were cyclical. A previous study (24) collected questionnaires from blind subjects in France (*n* = 794) in which over 83% reported at least one sleep problem (long sleep latency, nighttime or early morning awakenings, non-restorative, and poor sleep quality) vs 57% of controls, and 18% of the blind subjects vs 8% of controls having clinical criteria for non-24 SWRD. In a survey of 388 blind subjects, sleep disorders were reported by 48.7% of blind subjects compared to 9% in controls, and in up to 66% of those with a complete loss of light perception (25). Another observational survey conducted in a New Zealand blind community confirmed a higher incidence of sleep disorders, especially sleep-timing problems in those with reduced light perception (55%) vs 4% of the matched normal general population (26).

Overall, these studies confirm that sleep disorders are common among blind subjects with no perception of light and that cyclical symptoms and problems with sleep timing are present in a high frequency up to 66% (25).

## Definition of Non-24-h Sleep–Wake Rhythm Disorder

A non-24 SWRD is defined by ICSD-3 criteria according to 4 criteria (27): 1/History of insomnia, excessive daytime sleepiness, or both, which alternate with asymptomatic episodes, due to misalignment between the 24-h light-dark cycle and the non-entrained endogenous circadian rhythm of sleep–wake propensity; 2/Symptoms persist over the course of at least 3 months, 3/Daily sleep logs and actigraphy for at least 14 days, preferably longer for blind persons, demonstrate a pattern of sleep and wake times that typically delay each day, with a circadian period that is usually longer than 24 h, and 4/the disorder is not better explained by another current sleep disorder, medical or neurological disorder, mental disorder, and medication use of substance use disorder.

Presentation is often with a progressive delay in the sleep–wake pattern accompanied by a progressive increase in sleep onset latency and delayed sleep onset. The symptoms of non-24 SWRD occur periodically when the intrinsic circadian rhythm and the 24-h social rhythm run out of phase with each other leading to marked symptoms (i.e., both daytime sleepiness and nighttime insomnia) when the circadian rhythm promotes sleep in the middle of the day. In addition to sleep–wake problems, other rhythms such as the melatonin and the cortisol secretion are leading to problems with other circadian functions such as appetite and digestion.

The frequency of symptomatic periods depends on the daily delay. The magnitude of the daily delay varies as a function of the endogenous circadian period from less than 30 min (i.e., when the period is close to 24 h) to more than 1 h (i.e., when the period is longer than 25 h). A patient with an intrinsic period of 25.2 h will delay his/her clock by just over an hour every day and, thus, be in phase with the solar cycle for a short period every 3 weeks. During this time, they will be asymptomatic but as their circadian rhythms continue to drift the symptoms will become more severe, peaking when their circadian rhythm is in opposition to the solar cycle, which will occur around 10 days later. As the period of the instrinsic clock varies widely between patients, the frequency of symptomatic periods is also variable and may vary from 3 to 26 weeks. Diagnosis is complicated by the high prevalence of non-circadian sleep disorders (i.e., insomnia, sleepiness), depression, and anxiety in blind subjects, which may also lead to variable sleep complaints. The measurement of circadian biomarkers is, thus, mandatory in the diagnosis of underlying circadian disorders in complex cases.

Measurement of the main melatonin metabolite, 6-sulfatoxymelatonin (aMT6s) is recommended to confirm non-24 SWRD (19, 22, 28) using 24 h urine, saliva, or blood samples repeated two to three times over 2–4 weeks. Urinary samples are typically collected every 4 h during the day and every 8–10 h overnight. Repeated sampling will show the periodicity of the circadian clock in the abnormal range (i.e., <23.8 or >24.2 h) and demonstrate gradual drifting of the melatonin secretion profile. However, repeated 6-sulfatoxymelatonin measurement is expensive and should be reserved for cases where there is a high probability of non-24 SWRD. Other biomarkers such as cortisol may provide alternative measures of circadian phase in complex cases or when melatonin secretion is abnormal: for example, after pineal resection.

Due to its rarity in the general population, non-24 SWRD remains poorly understood and patients often experience a long delay (i.e., several years) before diagnosis. This diagnostic delay is exacerbated by the lack of sleep physicians with knowledge of circadian disorders available to diagnose and manage the disease. As prevalence is much higher in blind patients, an 8-item pre-screening questionnaire for visually impaired subjects has been developed to help physicians (22) both in clinical practice and in research. However, the questionnaire only asks patients about symptoms over the past month and, as a result, patients whose frequency of symptomatic periods is longer than 1 month may screen negative. This should be borne in mind when interpreting the results. Patients who screen positive should be offered a clinical interview in order to confirm the typical cyclical pattern of symptoms, followed by actigraphy and a sleep diary over prolonged periods. However drifting in sleep–wake cycle by actimetry or sleep agenda is not always evident in active individuals, and diagnosis should be confirmed by biomarker measurement. Application of this screening procedure should avoid measuring circadian biomarkers in patients in whom the probability of non-24 SWRD is low.

## Studies of Non-24-h Sleep–Wake Rhythm Disorder in the Blind

The first description of non-24 SWRD was reported by Miles (29) in the case of a congenitally blind 28-year man with no conscious light perception who complained of insomnia and hypersomnolence interfering with work and leisure activities. An extensive study of his circadian rhythms *via* a complex protocol including periods with ad-lib sleep and entrainment (strict schedule of bedtime, meals, and activity), showed desynchronization of the circadian rhythms of body temperature, vigilance, performance, cortisol secretion, and urinary electrolyte excretion, with a 24.9 circadian period, explaining the drift of his circadian rhythms and cyclical presentation of symptoms.

Further studies of sleep disorders in blind subjects were reported by Sack and by Lockley (30, 31) reinforcing a link between sleep disorders and circadian desynchronization. Using serial 24-h samples of plasma melatonin and cortisol in blind people with sleep complaints, Sack et al. found increased recurrent insomnia and daytime sleepiness symptoms when the internal rhythms were out of phase with the preferred sleep times (32). A further study in blind subjects confirmed that abnormal 24-h urinary excretion of aMT6s was found in subjects having free-running naps of long duration compared to those with normal rhythms (31). Another measurement of 4-hourly sequential urine samples during 48 h each week for several weeks performed in blind individuals with and without light perception showed a correlation between aMT6s excretion and cortisol rhythms in the blind subjects, independently of the circadian rhythm phenotype (entrained or free-running) (33). Further work by Lockley et al. (34) established the role of circadian sleep disorders on waking function: alertness, reaction time, and mood. Comparing the sleep–wake timing, circadian phase, and aMT6s excretion in untreated blind subjects with and without light perception at home for 4 weeks, they found three phenotypes: normal aMT6s rhythms, abnormally entrained (i.e., advanced or delayed), and non-24 SWRD. All patients with non-24 SWRD had no light perception and presented waking performance variability as a function of the circadian phase with higher sleepiness during the time of peak melatonin production. This contrasted with other blind subjects in whom vigilance, mood, and performance deteriorated significantly with increased time awake. A more recent study used daily sleep diaries and sequential measurement of 6-sulfatoxymelatonin urinary excretion over 48-h on two to three occasions separated by at least 2 weeks in 127 blind women with and without light perception (35). Among the 41 participants without light perception, 63% had an abnormal phase (24% advanced or delayed), 39% had non-24 SWRD and 37% had normally entrained rhythms. By contrast, among the 86 participants with light perception, 69% were normally entrained, 21% had an abnormal phase (advanced or delayed), and only 9% had non-24 SWRD.

All these results showed a striking relationship between blindness and abnormal circadian rhythms. Melatonin secretion during the day due to abnormal circadian rhythms is associated with daytime naps, strongly suggesting that abnormally timed endogenous melatonin induce hypersomnolence in blind subjects. The internal phase relationship between sleep–wake cycles and the circadian melatonin rhythm in entrained subjects leads to differences in the daily profile of vigilance, mood, emotion, and performance. Disruption of such relationship in blind individuals with non-24 SWRD resulted in impaired functioning in cerebral wake systems during the day.

Altogether, the frequency of sleep disturbances in blind individuals is close to 60–80% and over half of totally blind patients may have non-24 SWRD. The prevalence, gender, and ethnic differences of non-24 SWRD in adults and children remain unknown. The natural course of non-24 SWRD remains to be explored: onset is not always synchronous with total loss of vision, occurs in both congenitally blind children and adults, and the evolution of symptoms over time is unclear. The frequency of this condition in sighted people is unknown but certainly extremely rare even in the context of traumatic brain injury, developmental intellectual disability (Rett syndrome, autism spectrum disorders, Angelman syndrome), dementia or severe mental illness (example of schizophrenia) (36). In these patients, non-24 SWRD may be due to congenital or acquired lesions of the circadian systems, to reduced activation of intact neuronal circuits by circadian synchronizers or to reduced exposure to circadian synchronizers.

## Treatment of Blind Patients with Non-24 h Sleep–Wake Rhythm Disorder

Treatment of totally blind patients suffering from non-24 SWRD is considered effective when circadian rhythms are entrained to a normal 24 h cycle. Once rhythms are entrained, the patient remains synchronized with a normal 24-h day and the periodic symptoms of insomnia and excessive daytime sleepiness disappear.

First-line management consists of non-drug therapies, which aim to reinforce alternative zeitgebers (synchronizers of circadian rhythms). Patients are encouraged to set regular bedtime, getting up times, and meal schedules to participate in physical activities in the morning. If some light perception persists, daylight or bright light exposure in the morning is recommended. Stimulation of the wake systems by intellectual activities, cold showers, or intense physical exercise in the morning may be helpful.

Drug therapy includes rapid release melatonin (available without prescription in many countries), melatonin prolonged release, and melatonin agonists. Initiation of treatments may be most effective when the patient is in phase with the solar cycle as the aim of treatment is to prevent further circadian drift. It may be necessary to wait until the patient’s bed and wake times are approximately normal. However, this may not always be possible.

## Exogenous Melatonin Administration

Melatonin has a short half-life, ranging from 20 to 45 min (37), being largely due to an extensive hepatic first-pass metabolism. The role of melatonin on circadian rhythms needs to differentiate its acute sedative effects from its phase-shifting effects also referred to as “chronobiotic” effects (38). Exogenous melatonin treatment has both acute sedative and phase-shifting effects on sleep and other strongly endogenous circadian rhythms such as core body temperature; however, its shifting effects are determined by the time of administration following a “phase-response curve” (30, 39). Melatonin given in the early evening advance the circadian clock leading to earlier falling asleep and waking times, while early morning medication intake delays the circadian clock with later sleep onset and waking times. However, melatonin is less efficient as a delayer of circadian rhythm when given in the morning (with risk of acute drowsiness) than an advancer of circadian rhythm when given in the early evening (40, 41). Accordingly, exogenous melatonin given in the late afternoon is often effective in delayed sleep phase syndrome where patients need to fall asleep and wake earlier than they are currently doing (42). By contrast, melatonin has little or no effect given in the early morning in advanced sleep phase syndrome where patients need to fall asleep and wake later than they used to. Taken in the middle of the day, there is no phase-shifting effect of melatonin and, thus, no meaningful clinical effects. In non-24 SWRD, the effectiveness of exogenous melatonin depends on the circadian phase of the patient when the treatment is initiated. If the subject is in phase or the phase is beginning to delay, evening melatonin will effectively advance the phase and appropriately entrain circadian rhythms.

Several studies have reported the effects of rapid release melatonin in totally blind patients with non-24 SWRD (28, 30, 43–48) and one study has looked at prolonged release melatonin (49) (Table 1). The majority of these studies included only few patients and were open or single blind (28, 43, 48, 50). The study of Roth et al. on prolonged release melatonin was randomized, double blind, and placebo controlled (49).

**Table 1:** Treatment of non-24-h sleep–wake rhythm disorder.

Authors	Patients	Type of study	Treatment	Duration of treatment	Sample size	Action	Grade of study
Lockley et al. (48)	Totally blind with non-light perception and non-24 h	Single blind	5 patients: melatonine 5 mg or placebo	From 35 to 71 days	7 patients	Entrained circadian rhythms in four over seven patients	Low
			2 patients: melatonine 5 mg vs baseline			Clinical results not reported	

Sack et al. (28)	Totally blind with non-light perception and non-24 h	Randomized double blind	Melatonine 10 mg or placebo	3–9 weeks	7 patients	Entrainment 6 over 7 patients	Moderate
		Cross over				Improvement of sleep	

Hack et al. (45)	Totally blind with non light perception and non-24 h	Single blind	Melatonin 0.5 mg or placebo	26–81 days	9 patients	Entrainment 4 over 9 patients	Low
						Improvement of sleep	

Roth et al. (49)	Totally blind with non-light perception	Randomized double blind 1:1	Prolong release melatonin 2 mg or placebo 1:1	6 weeks	13 patients	Improved sleep	Moderate

Lockley et al. (19)	Totally blind with non-light perception and non-24 h	Randomized doubled blind 1:1	Tasimelteon 20 mg or placebo for 26 weeks 1:1	26 weeks	84 patients	20% improved sleep and wake, and entrained after 1 month of treatment (SET study)	High
						90% maintained entrainment vs 10% in placebo group (RESET study)	

*Grade of study*.

*High: randomized study with large sample size significant results and no evidence of bias*.

*Moderate: some but not all of the above criteria*.

*Low: observational studies with small sample sizes*.

A small crossover study using 10 mg of rapid release melatonin vs placebo in seven patients (28) showed successful entrainment of circadian rhythms in 6/7 patients on melatonin and no patient on placebo which was reflected by higher sleep efficiency and less time spent awake during the night on polysomnography. Circadian entrainment was maintained on low dose 0.5 mg of melatonin/day over a period of 3–4 months. Entrainment of circadian rhythms is also possible with low-dose melatonin, although this seems to be less successful. A single blind study of nine patients given either melatonin 0.5 mg or placebo showed complete and rapid entraining of the circadian clock as measured by cortisol secretion profiles in four over 9 patients, slower entraining in two and a partial response in one (45). Rapid entraining was associated with commencing treatment at the moment when rhythms were aligned or slightly delayed. Circadian rhythms started to drift again when melatonin treatment stopped. Finally, prolonged release melatonin [Circadin(^®^)] has been shown to improve sleep duration compared to placebo in 13 blind patients which is consistent with a circadian effect (49). Melatonin and prolonged release melatonin are well tolerated.

## Melatonin Receptor Agonists: Tasimelteon

Several melatonin receptor agonists exist, but only Tasimelteon (Vanda Pharmaceuticals inc. Rockville, MD, USA), has been approved for the treatment of non-24 SWRD by the FDA and the European Medicines Agency (EMA). Tasimelteon is a potent and specific melatonin (MT1 and MT2) receptor agonist with two to four times greater affinity for the MT2 receptor. Peak plasma concentration is reached 0.5 to 3 h after administration with a half-life of 1.3 h. Tasimelteon has been shown to affect circadian phase in healthy individuals (51). Two trials in totally blind patients with clearly defined non-24 SWRD showed that only 84/391 (22%) of screened patients had a circadian period of over 24.25 h (19). Treatment with tasimelteon or placebo was timed to coincide with the moment when rhythms (measured by urinary 6-sulfatoxymelatonin) were slightly delayed and given 1 h before target bedtime. After 1 month 8/40 (20%) of the tasimelteon group and 1/38 (3%) in the placebo group were entrained and a clinical result (less daytime sleep and increased nighttime sleep during the worst periods) was seen in 9/38 of the tasimelteon and 0/34 of the placebo groups (Table 1). A further period of ongoing treatment, including new patients with a shorter circadian period showed that entrainment was maintained in 9/10 (90%) of the tasimelteon group vs 1/10 (10%) in the placebo group. Tasimelteon was shown to be well tolerated over the short term although headache (17 vs 7% with placebo), elevated liver enzymes (10 vs 5%), nightmares or abnormal dreams (10% vs none), upper respiratory tract (7% vs none), and urinary tract infections (7 vs 2%) were more common. Long-term tasimelteon administration seems safe and well tolerated: a pooled safety analysis of the six clinical trials by Leger et al. (52) showed a total exposure of 258.64 patient years of use and similar discontinuation rates across tasimelteon and placebo.

## Conclusion

The master clock plays a vital role in the regulation of the circadian rhythms of several major biological processes. Its natural period is slightly longer than 24 h and requires daily synchronization with the solar cycle by exposure to morning light. Melatonin is important in the regulation of the circadian rhythms: its production is inhibited by light and a feedback loop favors circadian synchronization. In totally blind people, the absence of light impairs circadian synchronization, and in some this leads to gradual drift of their circadian rhythms following the underlying period of their biological clock. This gradual desynchronization leads to non-24 SWRD characterized by cyclical periods of severe insomnia and excessive daytime sleepiness maximal when a patient’s circadian phase is in opposition to the solar day. The resulting social and professional handicap may be severe and lead to social isolation and psychological difficulties. Rare in the general population and considered an orphan disease, non-24 SWRD is common in the totally blind. Diagnosis requires demonstration of a drifting sleep–wake cycle using sleep diary or actimetry and can be confirmed by repeated measurement of circadian biomarkers such as melatonin. Treatment to entrain and maintain circadian rhythms is effective and should combine behavioral and drug approaches. Treatments that have been shown to be safe and effective in this disorder include melatonin and the melatonin agonist Tasimelteon. Tasimelteon is to date the only treatment approved by the FDA and the EMA for non-24 SWRD in totally blind persons.

## Author Contributions

MAQS and YD reviewed all the literature and wrote the first draft of the manuscript. DL and SH revised the manuscript.

## Conflict of Interest Statement

YD received funds for seminars, board engagements, and travel to conferences by UCB Pharma, Jazz, Theranexus, Actalion, Vanda, Takeda, Flamel, and Bioprojet. DL is or has been consulted as the main investigator in studies sponsored by Actelion, Agence Spatiale Européenne, Ag2R, Bioprojet, CNES, DGA, ESAI, iSommeil, Jazz, Vanda, Merck, NASA, Philips, Resmed, Sanofi, Rhythm, Vinci Fondation, and Vitalaire in the last 5 years. MS received funds for seminars, board engagements, and travel to conferences by IRIS, BIOCODEX, and VANDA. SH received funds for conference participation from SOS Oxygène.
